# Implementation opportunities and challenges to piloting a community-based drug-checking intervention for sexual and gender minority men in Vancouver, Canada: a qualitative study

**DOI:** 10.1186/s12954-024-01004-y

**Published:** 2024-04-27

**Authors:** Pierre-julien Coulaud, Koharu Loulou Chayama, Cameron Schwartz, Aaron Purdie, Mark Lysyshyn, Lianping Ti, Rod Knight

**Affiliations:** 1 British Columbia Centre on Substance Use, Vancouver, BC Canada; 2 Department of Medicine, University of British Columbia, Vancouver, BC Canada; 3 Interdisciplinary Studies Graduate Program, University of British Columbia, Vancouver, BC Canada; 4 Community-Based Research Centre, Vancouver, BC Canada; 5 Health Initiative for Men, Vancouver, BC Canada; 6 Vancouver Coastal Health, Vancouver, BC Canada; 7 School of Population and Public Health, University of British Columbia, Vancouver, BC Canada; 8 École de Santé Publique de l’Université de Montréal, Montréal, Québec Canada; 9 Centre de recherche en santé publique (CReSP), Montréal, Québec Canada

**Keywords:** Drug checking, Community-based approach, Consolidated framework for implementation research, Sexual and gender minority, Qualitative study, Canada

## Abstract

**Background:**

In response to the overdose crisis, a collaborative group of two community-based organizations, a health authority and a research institute in Vancouver, Canada, implemented a pilot community-based drug checking (CBDC) intervention for sexual and gender minority (SGM) men. This study identified key factors that influenced the implementation of the CBDC intervention, including opportunities and challenges.

**Methods:**

We conducted semi-structured interviews with seven pertinent parties involved in the CBDC, including policymakers, researchers and representatives from community-based organizations. These interviews were coded and analyzed using domains and constructs of the Consolidated Framework for Implementation Research.

**Results:**

While drug-related stigma was identified as a challenge to deliver drug checking services, participants described the context of the overdose crisis as a key facilitator to engage collaboration between relevant organizations (e.g., health authorities, medical health officers, community organizations) to design, resource and implement the CBDC intervention. The implementation of the CBDC intervention was also influenced by SGM-specific needs and resources (e.g., lack of information about the drug supply). The high level of interest of SGM organizations in providing harm reduction services combined with the need to expand drug checking into community spaces represented two key opportunities for the CBDC intervention. Here, SGM organizations were recognized as valued partners that fostered a broader culture of harm reduction. Participants’ emphasis that knowing the composition of one’s drugs is a “right to know”, particularly in the context of a highly contaminated illicit drug market, emerged as a key implementation factor. Lastly, participants emphasized the importance of involving SGM community groups at all stages of the implementation process to ensure that the CBDC intervention is appropriately tailored to SGM men.

**Conclusions:**

The context of the overdose crisis and the involvement of SGM organizations were key facilitators to the implementation of a drug checking intervention in SGM community spaces. This study offers contextualized understandings about how SGM knowledge and experiences can contribute to implement tailored drug checking interventions.

**Supplementary Information:**

The online version contains supplementary material available at 10.1186/s12954-024-01004-y.

## Background

North America is currently in the midst of an overdose crisis. In Canada, opioid overdose accounted for 18.8 deaths per 100,000 in 2022, with the province of British Columbia (BC) experiencing 44.0 deaths per 100,000 [1]. The main driver of the high fatal overdose rate is the proliferation of illicit synthetic opioids (e.g., fentanyl/analogues) and fentanyl adulteration in stimulant and other non-opioid drugs poisoning street drug supplies across North America [2–7]. In response to the overdose crisis, several interventions have been implemented and upscaled including overdose prevention sites [8, 9], naloxone training and distribution [10–12] and supervised drug consumption services in various settings (e.g., housing, hospitals, public spaces) [13–17]. Despite the implementation of these interventions, fatal overdose rates continue to rise throughout various jurisdictions in North America [4, 18], thereby underscoring the urgency to develop novel harm reduction interventions.

Recently, drug checking services – a harm reduction strategy that aims to provide personalized, fact-based information regarding the composition of substances (including potential contaminants) to people who use drugs (PWUD) [19–21] – have been identified as a potential intervention to help reduce fatal overdose rates amidst the overdose crisis [22]. Drug checking provides the opportunity for PWUD to make more informed decisions about substance use and receive counseling and harm reduction education, as well as referrals to other drug-related services (e.g., substance use disorder treatment, naloxone training) [23]. Drug checking can be delivered on-site (e.g., at a festival, nightclub) or in a fixed-site service (e.g., supervised injection service) to serve as a point of entry for PWUD and other community members (e.g., family, friends, suppliers) to access other health information and services (e.g., primary care, social support programs) [24–26]. There are a variety of drug checking technologies in use with varying degrees of accuracy, usability, and costs [20, 27–30], ranging from low specificity and sensitivity at low price (e.g., colorimetric reagents, fentanyl test strips) to highly sophisticated technologies that are able to detect multiple compounds in a short period of time at relatively high costs (e.g., high-performance liquid chromatography, mass spectrometry).

Preliminary research on drug checking interventions has provided helpful information regarding the acceptability of drug checking among PWUD [31, 32], the ability to generate critical safety information and facilitate drug supply monitoring [21, 33], and the influence on drug use-related behaviour, including the intention to not use, reduce the dose, or discard the analyzed substance when the result is unexpected [29, 34–36]. Beyond these outcome measures, the implementation of drug checking interventions is complex and requires significant investments to be effective [37, 38]. For example, the context of drug criminalization and stigmatization, the costs associated with advanced technologies (e.g., maintenance) as well as the difficulty of navigating an unpredictable drug market were described as key challenges in implementing drug checking services in North America [39, 40]. While the legislative context regarding illicit drugs remains a major barrier to implementing drug checking services (e.g., at risk of criminal prosecution for staff persons and users) [38, 40], some jurisdictions have shifted their legal framework, moving from enforcement-led approaches towards public health-oriented policies to support the deployment and funding of these services [41, 42]. Several facilitators have also been identified such as the support of health authorities, the development of trusted relationships with providers, and the involvement of PWUD in the design and delivery of drug checking services [23, 39, 43]. While this body of evidence has been helpful for informing the implementation of drug checking interventions for some groups of PWUD (e.g., people who inject drugs) [21, 35], less attention has focused on how drug checking can be adapted to address the needs of other key populations, including sexual and gender minority (SGM) men who use drugs [44, 45].

SGM men represent a population that is potentially poised to benefit from drug checking services, as previous evidence highlights how SGM men have higher rates of both opioid and non-opioid substance use than their heterosexual and/or cisgender counterparts [46]. Furthermore, while previous research has identified how SGM people used a large variety of substances (e.g., opioids, stimulants, hallucinogens) with diverse motivations (e.g., enhance social connection, reduce anxiety and stress) in different settings (e.g., bars, nightclubs) and contexts (e.g., before or during sex, during transitions from adolescent to young adulthood) [47–51], research examining how harm reduction interventions, including drug checking services, can address drug-related harms among SGM people remains somewhat elusive [44, 45, 52]. To respond to this knowledge gap, this qualitative study aims to identify the key opportunities and challenges that influenced the implementation of a recent community-based drug checking (CBDC) pilot intervention that was specifically designed for SGM men in Vancouver, Canada. Specifically, our data collection and analysis draw on the Consolidated Framework for Intervention Research (CFIR) to provide a comprehensive understanding of various contextual factors that may affect CDBC intervention implementation in the real world [53].

## Methods

### Setting

In the Canadian province of BC, drug checking services have historically been unsanctioned (i.e., offered without authorization from the state). The first drug checking services developed as a harm reduction intervention in BC were operated by non-profit organizations in the late 1990s to provide peer-based colorimetric reagent testing for young people attending music festivals [54]. Drug checking interventions were often carried out by volunteers with a risk of legal consequences, increasing the challenges of accessing and implementing drug checking services for drug users (e.g., charges related to drug possession) and organizations (e.g., charges related to aiding or abetting illicit drug use) [55]. Since early 2000’s, the AIDS Network Kootaney Outreach and Support Society (ANKORS) provided on-site drug checking services at the Shambhala music festival, offering colorimetric reagent tests alongside other harm reduction supplies [34]. Since the mid-2010’s, the significant increase in drug-related overdoses and deaths has resulted in a radical change in the way that BC’s health authorities conceive drug checking. Following the provincial declaration of a public health emergency related to overdose deaths in April 2016 [56], drug checking has been recognized not only as an intervention to monitor the continuously shifting illicit drug market but also as an integral component of a comprehensive harm reduction approach. This legislative shift allowed ANKORS to support the deployment of more sophisticated drug checking technologies at music festivals (e.g., spectrometer) and mobile thin layer chromatography kits to accommodate with the increasing demand [28]. However, most drug checking services available in BC (fentanyl test strips) have been implemented for people who inject drugs in Vancouver’s inner-city neighborhood of the Downtown Eastside and in Downtown Victoria (including in supervised injection and overdose prevention sites, pharmacies), which limits access for other local communities who do not inject drugs [21, 35, 39].

### Community-based drug checking pilot intervention

**Fig. 1:**
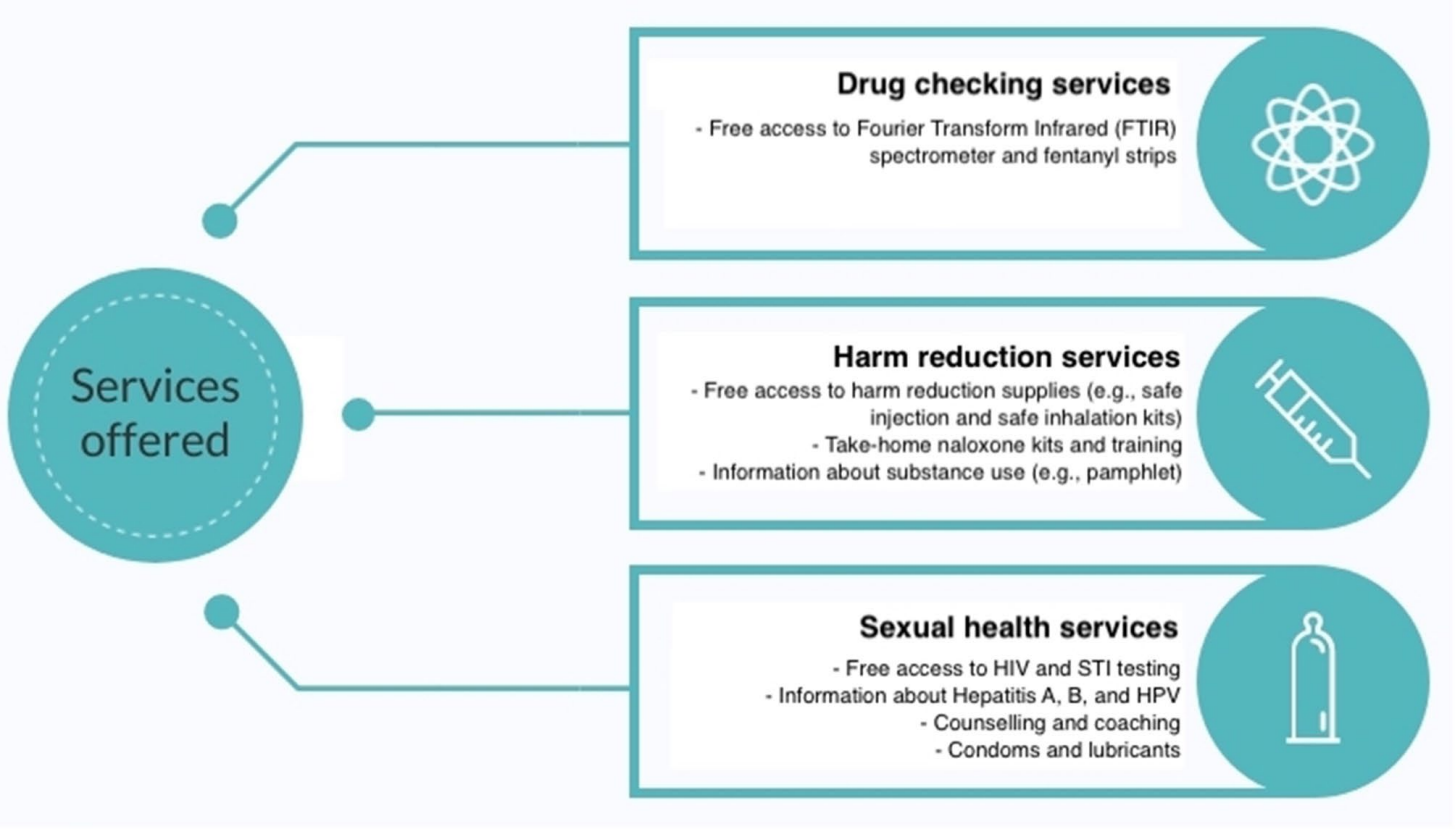
Description of the services offered during the community-based drug checking pilot intervention

From July 2018 to March 2019, a CBDC pilot intervention was initiated by a collaborative group including two community-based organizations, a research institute and a regional health authority to provide drug checking service for SGM men. These organizations had regular meetings that involved SGM peers and PWUD to design and monitor the CBDC pilot intervention. The intervention was implemented in three phases and largely took place in a community health clinic located in Vancouver that offers sexual health services tailored for SGM men including services for HIV prevention, care and support. The first phase was over a week-long period leading up to Vancouver’s pride festival (between July and August 2018), and included three “pop-up” events: one at a bathhouse frequented by SGM men and two at the aforementioned health clinic. The second phase occurred between November and December 2018, and consisted of providing drug checking service once a week at the health clinic. The third phase of the pilot took place once every two weeks between January and March 2019. In addition to drug checking services, SGM men could also access safer sex materials (e.g., condoms, lubricants), harm reduction materials (e.g., safer injection kits, pipes), naloxone kits and/or training, as well as information on substance use and sexual health (Fig. 1). At the community health clinic, SGM men could access a rapid test via fentanyl immunoassay strips delivered by a trained volunteer, and/or a Fourier Transform Infrared spectrometer (FTIR) operated by a trained technician.

### Conceptual framework

To identify key factors that influenced the implementation of the CBDC pilot intervention, we draw on the CFIR that was developed to guide design, evaluation and implementation of evidence-based interventions [53]. Specifically, the CFIR features 39 constructs within five key domains related to the intervention’s implementation (i.e., Intervention characteristics, Outer setting, Inner setting, Characteristics of individuals, and Implementation process) [57]. Given the intervention was a pilot study designed to assess the feasibility of implementing drug checking service in a sexual health clinic, our interview guide was not designed to address all of the CFIR constructs; instead, we focused our interviews broadly on the five key CFIR domains (see interview procedures for more details).

### Study sample

Stakeholders (called hereafter pertinent parties due to the racist etymology of the term stakeholder, c.f [58]). who were involved in implementing the CBDC pilot intervention were invited by e-mail and all agreed to participate in this study. We adopted a snowball sampling strategy that focuses on deliberately recruiting pertinent parties across the relevant organizations involved who, by virtue of their various experiences and contributions to the CBDC pilot, have the capacity to reflect on the key opportunities and challenges that influenced the implementation of this intervention. The research protocol was approved by the University of British Columbia Providence Health Care Research Ethics Board (H16-01915).

### Interviews

In-depth, semi-structured individual interviews were conducted after the CBDC pilot intervention between March and July 2019. Interviews were conducted in English in our private office space by a researcher (co-author RK) and a research assistant, two cisgender men with extensive experience conducting qualitative interviews and who are members of the SGM community. Each interview was audio-recorded and lasted about 50 minutes. Before beginning the interview, all participants were informed about the study objectives and provided their written informed consent. Field notes were also taken during the interviews.

We designed our interview guide based on the five CFIR domains to identify barriers and facilitators relevant to the CBDC intervention. First, pertinent parties were asked about the relative advantage of implementing such intervention to SGM men (e.g., why was it important to have drug-checking services situated within a service that is focused on the needs of SGM men?) and the decision-making process on the choice of drug checking technologies. Second, participants were asked about social and structural factors that influenced the intervention implementation, including how the current overdose crisis and issues related to the unregulated drug supply (e.g., adulteration with fentanyl) shaped the development of the CBDC intervention. Third, participants were asked about organizational setting including relationships between organizations, internal buy-in, and how drug checking aligned with the values and priorities of the organizations. Fourth, participants were asked about the key players and individual skill sets that influenced the intervention implementation (e.g., who were the key influential individuals to get on board with this implementation and why?). Fifth, participants were asked about the discussions and reflections that occurred during the process of implementing the CBDC intervention.

### Data analysis

Interview recordings were transcribed verbatim, checked for accuracy and then uploaded to NVIVO 12 software. An abductive thematic analysis, which combines inductive and deductive approaches, was employed to analyze interviews [59, 60]. Each transcript was first hand-coded by the principal author to create a preliminary set of themes (“why”, “who”, “where”, “how”, “what”) to explore the characteristics of the implementation. Using an inductive approach, we generated specific sub-themes within and across the initial themes, with a particular emphasis on identifying the challenges and opportunities regarding the implementation of the CBDC intervention. Lastly, we used a deductive approach to classify these sub-themes according to the main constructs of the five CFIR domains. Only the constructs with the greatest influence on the implementation are reported in the results.

The reporting of this study followed the Consolidated criteria for reporting qualitative research (COREQ) (see supplementary file).

## Results

### Overview

A total of seven pertinent parties were interviewed (Table 1), including two policy makers, two participants working at a research institute (one manager and one service provider), and three representatives from community-based organizations at different levels of decision making (one supervisor, one manager, and one student intern). Participants’ experiences and perspectives relating to the pilot CBDC intervention were organized according to the five CFIR domains.

**Table 1:** Characteristics of participants

Pseudonym	Organization	Role in the pilot intervention
*Ben*	Community-based organization	Manager
*Bluewhale*	Health authority	Service provider
*Christa*	Research institute	Manager
*David*	Health authority	Advisor
*Drew*	Community-based organization	Supervisor
*Flamingo*	Research institute	Service provider
*Jake*	Community-based organization	Proposed project

### Outer settings: “this is kind of a chink in the window”

Throughout the interviews, participants described how *external policies and incentives* played an important role in the implementation. Specifically, participants described how the overdose crisis and the public health emergency declared by the BC government in 2016 changed the landscape of harm reduction interventions, prompting drug checking services to be rolled out in partnership with health authorities across BC. Participants acknowledged that the organizations solicited for support in the implementation of the CBDC intervention, such as health authorities, medical health officers, and police departments, were well informed on drug checking services, and, in turn, co-operative, as Christa described:

I think that it [the overdose crisis] has given us an opportunity to do drug checking. Drug checking’s something that people have been talking about for a long time, but legally has not been a possibility. And this is kind of a chink in the window. Like we can do it now … because of the opioid overdose crisis.

Participants described that the devastating impacts of the overdose crisis served as a facilitator to promote this pilot intervention, as Ben explained:

I think [the CBDC intervention] got a lot of traction, because of the current opiate crisis and the extreme overdoses. Any sort of media around the coverage of these topics, I think it’s going to get picked up, especially if it’s offering a sort of new service in hopes of combating it.

Despite this, a subset of participants identified challenges that they associated with the outer setting that was linked to drug criminalization and stigma. For example, Flamingo expressed how drug-related stigma served as a “moral barrier” to accessing drug checking services:

I think a lot of people who use drugs casually or infrequently, have a hard time considering themselves a drug user. So, to actually go out of your way to a site like that, to get your drugs checked, it’s kind of like a moral barrier, where you’re like, “I am identifying myself as someone who uses drugs, and I’m going to go out of my way to find out what’s in my drugs rather than just continue to kind of play dumb,” right?

Participants also described how drug-related stigma created challenges for staff who supported the delivery of this intervention. For example, Bluewhale expressed fear of judgement and described how a drug checking job could negatively impact their career advancement:

Yeah, it was nerve-wracking. I think that I was really nervous about like providers seeing me. As a volunteer, I was nervous of judgment, or career growth, from that. I actually had just applied for a drug checking job, and then of course one of the nurses who knew that I applied was there, and saw me, and they’re going to think that I’m using drugs.

Participants also acknowledged the importance of implementing an intervention that accounts for SGM-specific *needs and resources* – particularly with regards to the specific risks associated with the drugs and drug supplies that SGM people rely on and use. For example, participants described how our lack of understandings about the quality and risks associated with the drug supplies SGM people are accessing represented a key motivator to implementing the CBDC intervention. For example, Drew explained how drug checking data could be instrumental in our efforts to better understand the SGM-specific drug supply:

I think another factor that was interesting to us was sort of wondering and thinking about how the queer drug supply is or is not different from the drugs that other communities are getting. So, we were curious to answer the question, do we have issues with fentanyl contamination? What is the sort of the quality or consistency of the substances that are being used by queer guys? And so, there were lots of contributing interests, that made it like a good fit.

As a result of the overdose crisis, the willingness of health authorities and community-based organizations to expand drug checking for SGM men was a critical factor for designing and implementing the pilot CBDC intervention. The importance of addressing the needs of SGM men, a population with limited access to harm reduction interventions, was also a key facilitator.

### Intervention characteristics: “the more services the better”

Participant accounts elucidated how the *relative advantage* of implementing the intervention (i.e., participants’ perceptions of the need for an intervention) created opportunities for implementation. Participants described how the CBDC intervention was considered as an opportunity for health authorities to expand drug checking services in response to the overdose crisis. As a preventative measure against overdose, drug checking was regarded as an important harm reduction strategy to enhance knowledge among PWUD about the contents in their drugs, as Jake described:

I think part of it, for sure, was to decrease the chance of overdose […]. Just as like a primary harm reduction strategy, is just to make people aware of what’s in their substances, but also to prevent like other sorts of impurities like a bad experience.

During our interviews, participants also identified the *adaptability* of CBDC as a key intervention characteristic, particularly as this pertained to the ability to adapt the intervention to address SGM-specific needs. Participants explained that existing drug checking services, many of which at the time of the interviews were concentrated in the Downtown Eastside of Vancouver, were not tailored for those who used drugs recreationally, as Christa described:

There’s a large number of other people who are using drugs recreationally, who are not super comfortable going into those sites [supervised consumption and overdose prevention sites], and we don’t want to impose on those sites, because they’re very busy and focused on a certain clientele. So, we’d like to expand the number of drug checking sites.

As such, participants described how this gap in existing services led to an impetus to adapt a drug checking intervention to meet the needs of SGM men. Therefore, the CBDC pilot intervention was delivered outside of the Downtown Eastside and in community spaces relevant to SGM people, as Ben described:

… it’s really one of [health authority]’s new sort of attempts at solving the overdose crisis, right, around trying to get drug checking into community spaces, especially outside of sort of places in the Downtown Eastside where drug checking happens a lot, ….

Finally, participant accounts highlighted how *intervention source* influenced implementation. Specifically, the perception that the CBDC intervention was being or could be developed ‘from within’ (e.g., a grassroots approach featuring SGM voices and experiences) conferred significant legitimacy to the intervention. For example, participants described how the involvement of the design of the CBDC intervention featured the close involvement of a young cisgender gay man with experience delivering drug checking service as a volunteer at a music festival, as Christa explained:

There was one student there who had a ton of information about drug use in the population of gay men. […] Without the expertise of those community groups, it wouldn’t have been something that we would have ever thought about.

Participants also emphasized the high level of interest among community-based organizations serving SGM people (hereafter referred to as SGM organizations) in providing harm reduction services to their community given that these services were limited. For example, Drew described how this facilitated the connection of a legitimate harm reduction intervention to the queer community that their organization serves:

Well, we sort of collectively acknowledged that probably the more services the better. […] There were a number of factors. One was specifically the position of our student, who happened to have connections and experience with drug checking, and knew what the services were. Because I think until that point, there hadn’t been very much of a connection between harm reduction drug checking services and the queer community […] We were mostly just community members interested in increasing access to services for our community members.

### Inner settings: “getting together to figure out how this program might work best”

The majority of participants described how *implementation climate* between the organizations involved (i.e., SGM organizations, research institute, and health authority) was crucial to shaping the implementation. Participant accounts revealed a strong interest and a shared receptivity of involved pertinent parties to develop and support this intervention. For example, Ben described:

I guess it was primarily those four bodies, the [health authority], two community groups and one research organization specifically around substance use. So folks who all sort of have different overlapping concerns about this issue but from different approaches, getting together to figure out how this program might work best. We had a sort of community dialogue.

Participants also emphasized how *networks and communications* across organizations were critical to optimizing the implementation of the CBDC intervention. Participants described that each organization had a well-defined role in the implementation process and had dedicated time to meet and exchange ideas about the intervention. Participant accounts highlighted how pertinent parties ensured meaningful participation of SGM organizations during the implementation process. For example, Christa explained that SGM organizations were perceived as essential, valued and knowledgeable partners in the implementation, with further resources and lessons learned shared from the health authority and research institute:

The planning was really smooth. Everyone was really eager to provide the service, and it was really easy to schedule meetings because everyone was making a lot of time for it, which was great. There was a lot of communication to making sure that everybody’s on the same page and everybody’s always brought up to speed about things, especially community groups. Because they’re doing a lot of work and they’re not necessarily being paid as much as other people. We think of them as valued partners as well, and that we’re not stepping on toes.

Participants described how the *culture* within the community health clinic that delivered the CBDC intervention influenced the implementation process. Specifically, the community health clinic was described as being a critical to the success of the intervention as their services emphasized harm reduction and destigmatizing approaches at each step of the intervention implementation. As such, participants described how the existing culture of health promotion and harm reduction developed by the clinic served as a facilitator in the implementation process, as Jake described:

They [community-based clinic] are obviously quite sex positive, and they’re accepting of harm reduction sort of as an approach to substance use for folks that would benefit from it over abstinence. […] Like they’re not just openminded, but I think they don’t necessarily stigmatize substance use in a way that, I don’t know, other places might more.

Participant accounts highlighted how *available resources*, including shared resources dedicated to training, positively influenced the implementation. Nevertheless, several participants emphasized how volunteers and technicians trained in harm reduction and drug checking technologies were essential to the delivery of the CBDC intervention and simultaneously represented resource-intensive supports and challenges. For example, Christa explained that the implementation of FTIR required a trained technician who could test drug samples in real time and interact with clients through a harm reduction approach:

We’ve been having a really hard time, because it’s also having that harm reduction ability and the ability with interface with clients. […] you have to be able to run, to match the sample with the libraries and look at spectra, and make analytical calls in the moment. I think the pressure can be quite high because you don’t want to tell somebody the wrong information. So it’s just been very challenging. When we do hire someone, they have about a two-day intensive in-office training, and then we require a minimum of 30 hours of shadowing with an experienced technician. And then on top of that, they have to pass a practical exam and a written exam in order for us to say, “Okay, we’re comfortable with you operating this FTIR.

### Characteristics of individuals: “You have the right to know”

Several participants described how pertinent parties’ *knowledge and beliefs about the intervention* were crucial to shaping the implementation of CBDC. Participant accounts highlighted how those involved in the intervention were knowledgeable about the drug use patterns and harm reduction needs of SGM people and placed a high degree of value on the pilot intervention. Bluewhale noted that SGM people are often recreational drug users and less familiar with harm reduction strategies: *“queer folks don’t appear to be maybe a part of the big community of drug users, or don’t have a lot of information around how to use specific drugs safely.”*

Given that data regarding drug overdose in SGM community are largely unavailable in Canada, pertinent parties described how the pilot intervention provided an important opportunity to inform SGM people about the contents of their drugs, as Christa described:

I think there was some conversations saying that LGBTQ people use drugs in a different way than other people who use drugs recreationally, and that there’s a lot of misinformation in that community, in terms of fentanyl. […] And we were saying like there’s not a lot of fentanyl in these substances, typically, but we don’t have a lot of data to go on yet. And I think that there was a lot of concern voiced at those meetings about fentanyl specifically.

Participants’ strong desire to advance the CBDC intervention was also driven by their values pertaining to harm reduction and their belief that PWUD have the right to know what they are consuming; as David emphasized:

I think drug checking for fentanyl is one thing, but drug checking as a whole, again, you have the right to know what’s in your drug. You have the right to know that what this person sold you. So, I think like a lot of people are not that worried about fentanyl, but they’re going to still come and check their drug because they want to know what they put in their body.

### Implementation process: “the intent was to learn”

Towards the end of the interviews, participants were asked to describe how the processes of implementation (*planning and engaging*) influenced the intervention. Given that this intervention was a pilot, the implementation process was described as a learning experience to understand what kind of drug checking intervention can be offered to this population; as Drew explained:

It was a pilot. So, the intent was to learn. But there were just definitely some lessons that came out of it that would inform how we could move forward. I think one of the things that I really noted through the experience was just how important informing people about what the service actually was.

Participants also provided insights on the *planning* of the CBDC intervention. Participants acknowledged that the decision-making process regarding the drug checking service delivery method was based on a set of implementation criteria (e.g., availability of material and human resources) with the aim of facilitating access to services for SGM people. Drew described that these conversations led to the design of an intervention with multiple outreach drug checking strategies:

We thought that it would be best, instead of just trying one drop-in event, to try different configurations to see if time or location might have an impact over access to the service? So, we also recognized that there were so many factors that could influence what the people were accessing. For instance, when were they acquiring the substances they were planning on using for Pride? When would they have them like on them and available to do the checking? How would they and when would they be able to find out about the service? So, we ended up working out through a number of factors like, availability of space, availability of the spectrometer, and the technician, and the volunteers. We decided to have three drug checking drop-ins.

Several participants described how *engaging* community groups in the intervention influenced the implementation process. Specifically, participants emphasized the importance of involving SGM community groups at all stages of the implementation process to ensure that the intervention is tailored to SGM men. For example, Christa underlined how SGM organizations played a critical role in implementing the FTIR in community sites:

I would say one issue to operating the FTIR, has been just the number of stakeholders involved. Because like we are providing the technology, the equipment and the staff, but then we have to operate in a community site. So we need to make sure that the community group is involved at all stages of planning, and making sure that they’re looped in. Like if the technician can’t make a shift for some reason, we have to make sure that they know and that their communications people are looped in.

## Discussion

**Fig. 2:**
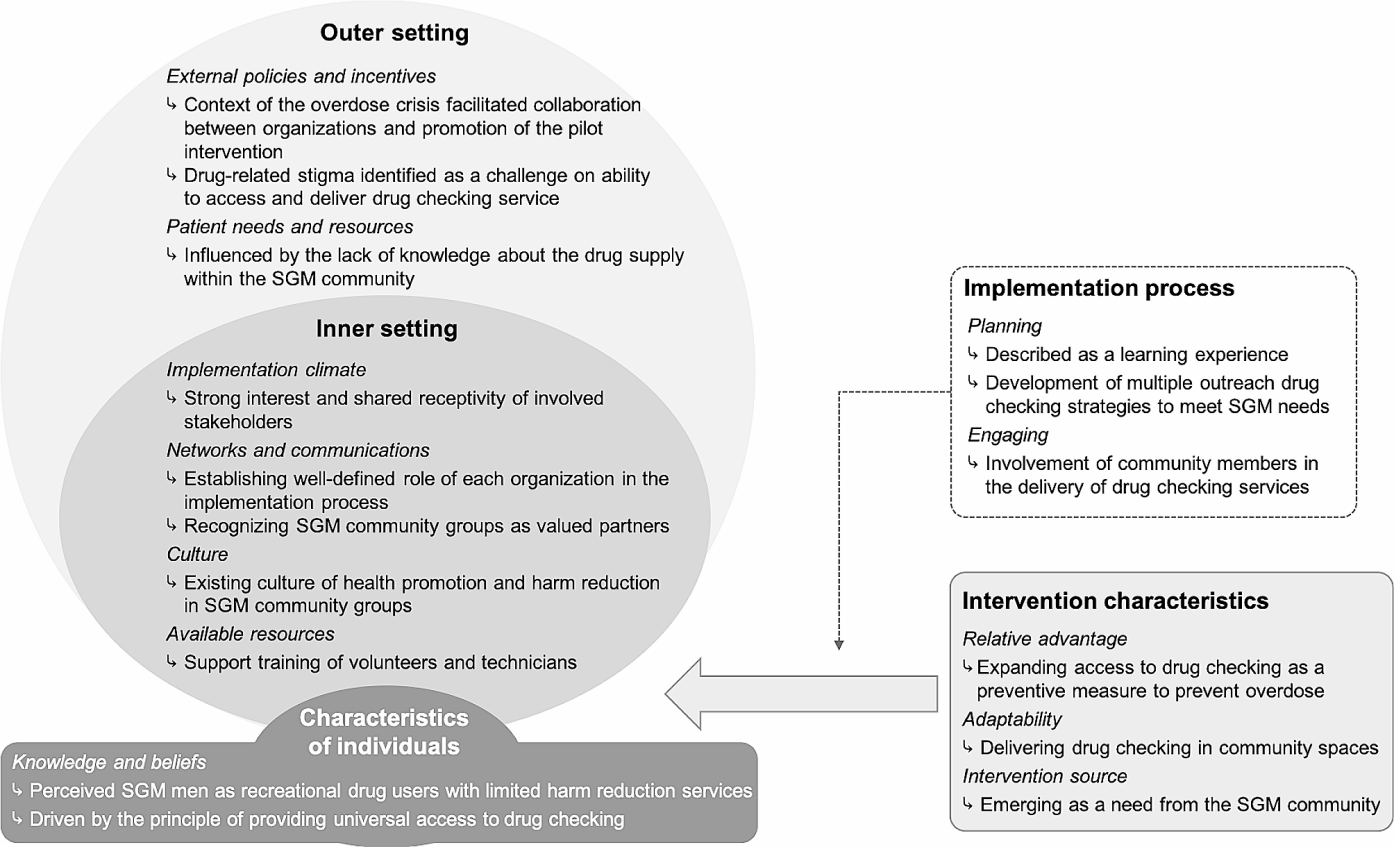
Summary on the main findings according to the Consolidated Framework for Implementation Research

Our findings highlight the challenges and opportunities associated with the implementation of a CBDC intervention for SGM men (see Fig. 2). Specifically, we found that the context of a public health crisis associated with overdose deeply impacted four of the five CFIR domains (except for *implementation process*). Participants described that the overdose crisis helped to mobilize support from relevant organizations (e.g., health authorities, medical health officers, police departments), and provide favourable conditions (e.g., legal framework) to deliver the intervention in sites relevant to SGM men (*outer and inner settings*). The overdose crisis also had an influence on the motivation for implementing the CBDC intervention, which included preventing overdoses and other drug-related harms within the SGM community (*intervention characteristics*). In addition, the *knowledge and beliefs* of pertinent parties emphasized the need to extend the reach of drug checking to a population at risk of potential drug contamination and overdoses based on values of harm reduction and a “right to know” for PWUD about what they are consuming (*individual characteristics*). These findings reflect the implementation of what Wallace et al.’s [61] characterized as a framework for proportionate universalism in drug checking. For example, our findings describe how the implementation of the CBDC intervention featured multiple tailored approaches that sought to address structural inequities and needs of SGM to maximize reach and access to drug checking services. Nonetheless, participants also discussed how drug-related stigma negatively influenced the implementation of the CBDC pilot intervention. As documented in previous studies [23, 39], drug-related stigma was identified as a barrier to service delivery for and access to CBDC for SGM men. Efforts to eliminate stigma, including decriminalization of drug use and possession, are needed to ensure successful implementation of CBDC.

Throughout our interviews, participants highlighted the importance of collaborating with SGM organizations in the implementation of the intervention. Drawing on community- and participatory-based approaches [62, 63], the CBDC pilot intervention was implemented using a bottom-up approach to promote community participation during the process of implementation and supported by policymakers and researchers (*intervention characteristics*). As such, this approach created opportunities for SGM organizations to provide critical information and knowledge about the substance use needs and resources that influenced pertinent parties’ perceptions (*individual characteristics*), including recognition as critical partners in the intervention implementation (*inner settings)*. Most importantly, involving SGM organizations also helped to overcome some challenges to optimize the *intervention process*, such as identifying community spaces and outreach strategies to deliver and promote drug checking (e.g., events during pride), as well as tailoring services by mobilizing SGM-specific community resources (e.g., health clinic premises, trained volunteers). Pertinent parties also emphasized the existing *culture* of harm reduction and health promotion within the SGM organizations as a key facilitator to implementation. These organizations have extensive experience navigating multiple forms of social and structural oppressions (such as norms of cis-heteropatriarchy), making them equipped to support the implementation of new interventions such as CBDC [64, 65].

This study has several strengths and limitations. First, our sample size was relatively small, with seven pertinent parties who were involved in the CBDC pilot intervention implemented in Vancouver. While we acknowledge that our study sample includes a relatively small number of participants, our sample represents a relatively exhaustive set of the pertinent parties involved in the implementation of this intervention that is suitable for in-depth qualitative inquiry to document the key opportunities and challenges that influenced intervention implementation. Second, the generalizability of our findings should be interpreted with caution, as our findings have focused on distilling context-sensitive insights, including a setting which the illicit drug supply is highly contaminated. These findings identified some of the crucial ingredients necessary in our local context (including the presence of a legal framework, political support, and engagement of local community organizations) for a community-based drug-checking intervention; however, other initiatives can use these findings as ‘clues’ for identifying the foundations on which their own community-based approaches can be used to implement local drug checking services. Third, our interview guide did not include questions to reflect all CFIR constructs, and therefore may have limited opportunities for participants to provide further insights regarding other constructs, such as *design quality*, *cost*, and *evaluating*. Lastly, we did not collect information on pertinent parties’ demographics, including whether any of the participants have lived experience as either PWUD or SGM. Future research should be conducted among SGM men to examine their experiences and perspectives on drug checking services.

## Conclusion

This study provides critical insights into the implementation of the first CBDC intervention for SGM men in Vancouver. Our findings describe how the context of a public health crisis (i.e., the overdose crisis) enhanced the level of supports provided by relevant organizations (e.g., health authorities, medical health officers, police departments) and provided favorable conditions (e.g., legal frameworks) for implementing the intervention. Furthermore, these findings underscore how the involvement of SGM organizations was critically important to intervention implementation, including identifying community spaces and outreach strategies to deliver and promote drug checking, as well as tailoring services by mobilizing SGM-specific community resources. Taken together, these findings underscore how community involvement in the implementation of future drug checking interventions for SGM will be critical to tailoring interventions appropriately.

### Electronic supplementary material

Below is the link to the electronic supplementary material.

Supplementary Material 1

## Data Availability

All relevant data are presented in this published article and are fully sufficient to replicate the study findings.

## Abbreviations

BC: British Columbia
CBDC: Community-Based Drug Checking
CFIR: Consolidated Framework for Implementation Research
FTIR: Fourier Transform Infrared spectrometer
SGM: Sexual and Gender Minority
PWUD: People Who Use Drugs
